# Nonalcoholic Fatty Liver Disease Is a Precursor of New-Onset Metabolic Syndrome in Metabolically Healthy Young Adults

**DOI:** 10.3390/jcm11040935

**Published:** 2022-02-11

**Authors:** Jeong-Ju Yoo, Eun Ju Cho, Goh Eun Chung, Young Chang, Yuri Cho, Sang-Hyun Park, Su-Min Jeong, Bo-Yeon Kim, Dong Wook Shin, Yun Joon Kim, Jung-Hwan Yoon, Kyungdo Han, Su Jong Yu

**Affiliations:** 1 Division of Gastroenterology and Hepatology, Department of Internal Medicine, Soonchunhyang University Bucheon Hospital, Bucheon 14584, Korea; puby17@naver.com; 2 Department of Internal Medicine and Liver Research Institute, Seoul National University College of Medicine, Seoul 03080, Korea; creatioex@gmail.com (E.J.C.); yoonjun@snu.ac.kr (Y.J.K.); yoonjh@snu.ac.kr (J.-H.Y.); 3 Department of Internal Medicine and Healthcare Research Institute, Healthcare System Gangnam Center, Seoul National University Hospital, Seoul 06236, Korea; gohwom@hanmail.net; 4 Department of Gastroenterology and Hepatology, Soonchunhyang University Seoul Hospital, Seoul 04401, Korea; chyoung86@gmail.com; 5 Center for Liver and Pancreatobiliary Cancer, National Cancer Center, Goyang 10408, Korea; presh_yuri@hanmail.net; 6 Department of Biostatistics, College of Medicine, Soongsil University, Seoul 06978, Korea; ujk8774@naver.com; 7 Department of Family Medicine & Supportive Care Center, Samsung Medical Center, Sungkyunkwan University School of Medicine, Seoul 06351, Korea; smjeong.fm@gmail.com (S.-M.J.); dwshin.md@gmail.com (D.W.S.); 8 Division of Endocrinology, Department of Internal Medicine, Soonchunhyang University Bucheon Hospital, Bucheon 14584, Korea; byby815@schmc.ac.kr; 9 Department of Clinical Research Design and Evaluation, Samsung Advanced Institute for Health Science, Seoul 06355, Korea; 10 Department of Digital Health, Samsung Advanced Institute for Health Science, Seoul 06355, Korea

**Keywords:** metabolic syndrome, nonalcoholic fatty liver disease, fatty liver index

## Abstract

Nonalcoholic fatty liver disease (NAFLD) is associated with metabolic syndrome (MetS). However, the temporal relationship between NAFLD and MetS has yet to be evaluated, especially in young adults. In this study, we investigated whether NAFLD could be a precursor for MetS in metabolically healthy young adults. Using the Korean nationwide health screening database, we analyzed subjects aged 20–39 years who were free from any component of MetS between 2009 and 2012. A total of 1,659,192 subjects without excessive alcohol consumption or concomitant liver disease were categorized into three groups according to the fatty liver index (FLI): (1) NAFLD (FLI ≥ 60); (2) borderline NAFLD (30 ≤ FLI < 60); and (3) control (FLI < 30). During the 6,699,462 person-years of follow-up, 109,239 subjects developed MetS (16.3 per 1000-person-years). The NAFLD group and the borderline NAFLD group were associated with a higher risk of MetS than the control group (incidence rate ratios, 2.9 (95% confidence interval (CI), 2.7–3.1) for the NAFLD group and 2.1 (95% CI, 2.1–2.2) for the borderline NAFLD group, respectively). In addition, all of the metabolic components were positively associated with FLI in a proportional manner. NAFLD is associated with the future onset of MetS in young adults. Therefore, active lifestyle intervention is required for young adults diagnosed with NAFLD to prevent MetS and other metabolic diseases.

## 1. Introduction

The prevalence of nonalcoholic fatty liver disease (NAFLD) is rapidly increasing, and NAFLD is associated with variable metabolic disorders, cardiovascular disease and even colonic diverticulosis [1,2,3]. Until recently, NAFLD was regarded as one of the manifestations of metabolic diseases [4]. However, in relation to NAFLD and diabetes mellitus (DM), it has been suggested that NAFLD may precede DM temporally, and that hepatic steatosis may be the cause of insulin resistance [5,6,7,8]. Therefore, we may presume that NAFLD is not a manifestation of metabolic diseases, but rather an early precursor of metabolic diseases [5]. Similar to the relationship between NAFLD and DM, NAFLD and metabolic syndrome (MetS) also have a strong association. Many cross-sectional studies have shown that the prevalence of NAFLD and MetS tends to be proportional [9]. Plus, several cohort studies have reported that NAFLD antedates several components of metabolic syndrome, such as hypertension and impaired fasting glucose [9,10,11,12]. However, most existing reports do not provide longitudinal temporal relationships due to the cross-sectional design, small number of patients, or short observation period of the studies [13,14]. Most researches lack long-term information regarding the antecedent relationship between NAFLD and MetS. In particular, few papers investigate the temporal relationship between the two diseases in a cohort study of young adults, a group where early intervention can reduce premature mortality or morbidity. Moreover, simple steatosis in liver has been reported to increase overall mortality, and cardiovascular-related mortality. Therefore, early intervention including a stricter lifestyle modification may be necessary to reduce NAFLD mortality [15].

In this study, we investigated whether NAFLD could antedate MetS in young adults that did not have MetS at baseline.

## 2. Materials and Methods

### 2.1. Data Source

We used the database of the Korean National Health Insurance System (NHIS), which is the national insurer managed by the Korean government. Approximately 97% of the Korean population subscribes to NHIS [16]. In Korea, national health screening is performed for employed workers, annually for blue collar workers, and biennially for white collar workers. The NHIS database contains annual or biennial health screening records for employees aged 40 or under, including sociodemographic data (age, sex, and income level), anthropometric measurements, laboratory tests (lipid profiles, blood glucose, etc.), questionnaires on lifestyle behaviors (smoking, alcohol consumption, and physical activity), medical diagnosis (based on the International Classification of Diseases, 10th revision (ICD-10)), and treatment data. This database has been widely used for various epidemiologic studies [17,18,19].

The Institutional Review Board of Seoul National University Hospital approved this study (IRB No, E-2012-106-1183). Informed consent was waived as only de-identified information was used for this study.

### 2.2. Study Population

**Figure 1:**
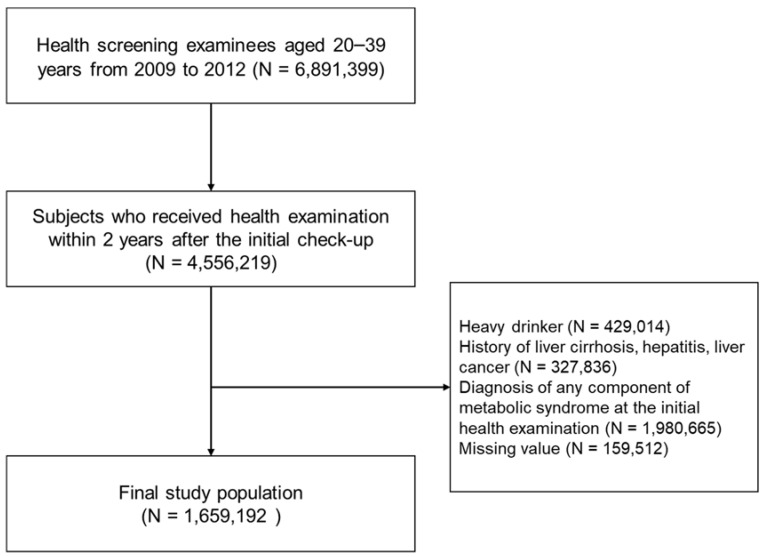
Flow chart of study population.

Subjects included in this study were adults aged 20 through 39 who underwent national health examinations between 2009 and 2012 (index year, considered as baseline), with subsequent annual or biennial health examination follow-ups until December 2018. Among the 6,891,399 subjects with health examination records in the index year, 4,556,219 who participated in at least one examination within 2 years after the initial check-up were included. Subjects with excessive alcohol consumption [20] (≥30 g/d for men and ≥20 g/d for women, N = 429,014), subjects with a history of chronic hepatitis, cirrhosis, or liver cancer before the index year (N = 327,836), those diagnosed with any component of metabolic syndrome at the initial examination (N = 1,980,665), and those with a missing value (N = 159,512) were excluded from this study. As a result, a total of 1,659,192 subjects were analyzed (Figure 1).

### 2.3. Data Collection

The following characteristics were collected at each health examination: age; sex; weight circumference (WC); body mass index (BMI); systolic and diastolic blood pressure; serum creatinine; total cholesterol; high-density lipoprotein (HDL) cholesterol; triglyceride; aspartate aminotransferase (AST); alanine aminotransferase (ALT); and fasting glucose levels. Blood samples were obtained from each participant after an overnight fast of ≥8 h. Lifestyle variables were obtained through questionnaires including the following information: smoking status (non-smoker, ex-smoker, or current smoker); alcohol consumption (none, mild-to-moderate drinking (less than 30 g/day for men, 20 g/day for women)); regular exercise (more than 20 to 30 min of moderate-to-vigorous activity at least three times per week). Income level was dichotomized at the lowest 20%. Comorbidities were defined using ICD-10 diagnostic codes, prescription information before the health examination, and blood test results.

### 2.4. Definition of NAFLD

Ultrasonography is not included in the NHIS health examination, and so the fatty liver index (FLI), which is a widely used alternative to imaging modalities in large epidemiologic studies [20], was used to assess hepatic steatosis and categorize subjects into three groups: (1) with NAFLD (FLI ≥ 60); (2) with borderline NAFLD (30 ≤ FLI < 60); and (3) without NAFLD (FLI < 30) [21,22]. To identify continuous trends, we also performed analyses based on deciles of FLI.

      FLI = e^0.953 × ln (triglyceride) + 0.139 × BMI + 0.718 × ln (gamma-glutamyl transferase) + 0.053 × WC − 15.745^/(1 + e^0.953^
^× ln (triglyceride) + 0.139 × BMI + 0.718 × ln (gamma-glutamyl transferase) + 0.053 × WC − 15.745^) × 100 (1)

### 2.5. Study Outcome

The study outcome was the occurrence of MetS defined by the harmonizing criteria [23], which requires at least three of the following components: (1) waist circumference [WC] ≥ 90 cm for men, or ≥85 cm for women; (2) triglycerides ≥150 mg/dL, and/or use of lipid lowering drug such as statin or fibrate; (3) HDL cholesterol <40 mg/dL for men, or <50 mg/dL for women and/or use of a relevant drug; (4) elevated blood pressure (systolic ≥130 mm Hg and/or diastolic ≥85 mm Hg) and/or use of an antihypertensive drug; and (5) fasting glucose ≥100 mg/dL, and/or use of an antidiabetic medication. The study population was followed from baseline to the health examination date at which MetS was first diagnosed or to the last available examination date. The average follow-up period was 4.04 (standard deviation, 2.29) person-years.

### 2.6. Statistical Analyses

Continuous variables are presented as means ± standard deviations (SDs), and as proportions for categorical variables, unless otherwise indicated. The Student’s t-test for continuous variables and χ^2^ test for categorical variables were used to analyze differences between the groups.

Regarding the time of MetS occurrence, it was assumed to be the midpoint between the examination date at which MetS was first diagnosed and its previous health examination date [24]. To estimate the risk of MetS, a series of multivariate Cox proportional hazard models adjusted for age, sex, smoking status, alcohol consumption, regular exercise, and BMI were constructed. Analyses were also performed by decile of FLI to identify continuous trends. The potential effect modification by age, sex, alcohol consumption, regular exercise, smoking status, obesity (BMI ≥ 25), and the presence of dyslipidemia was evaluated using stratified analysis.

Statistical analyses were performed using SAS version 9.4 (SAS Institute, Cary, NC, USA) and R version 3.2.3 (The R Foundation for Statistical Computing, Vienna, Austria, http://www.Rproject.org, accessed on 2 August 2021). A two-sided *p* value < 0.05 was considered statistically significant.

## 3. Results

### 3.1. Baseline Characteristics of the Study Population

Comparison of baseline characteristics according to the fatty liver index is shown in Table 1. Compared to the control group, the borderline NAFLD group and the NAFLD group included a higher percentage of older subjects (29.2 vs. 31.4 vs. 31.8 years old, *p* < 0.001) and males (43.3% vs. 95.9% vs. 96.8%, *p* < 0.001). The proportions of subjects with mild-to-moderate alcohol consumption, current smoking status, and regular exercise were higher in the borderline NAFLD group and the NAFLD group. Additionally, the borderline NAFLD and NAFLD groups were more likely to have dyslipidemia at baseline than the control group. Most anthropometric and laboratory profiles (including BMI, systolic and diastolic blood pressure, fasting glucose, and LDL and HDL cholesterol) were metabolically favorable in the control group compared to the borderline NAFLD group or the NAFLD group.

### 3.2. Incidence of Metabolic Syndrome and Its Component According to the Degree of Hepatic Steatosis

**Figure 2:**
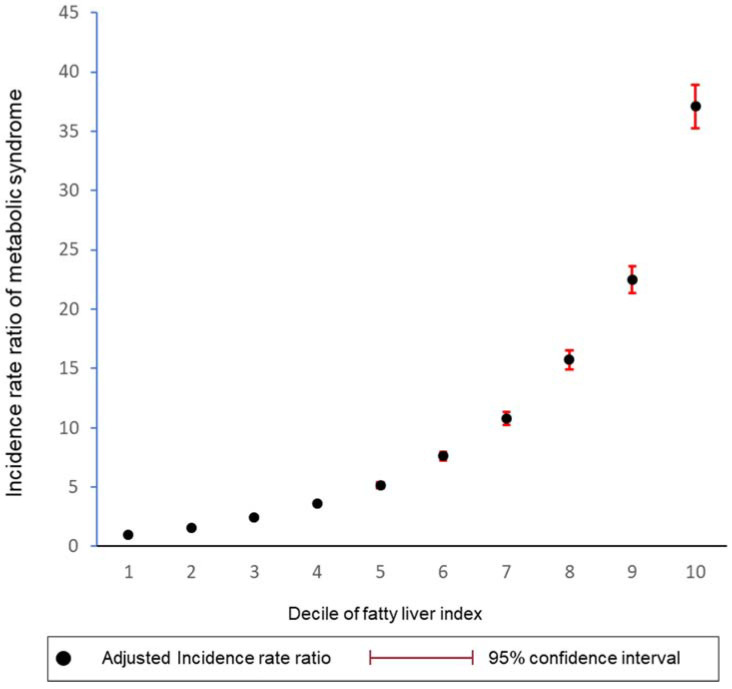
Fatty liver index and risk of metabolic syndrome by decile groups. The dot represents the incidence rate ratio, and the vertical line represents corresponding 95% confidence interval. The graph was adjusted for age, sex, alcohol consumption, smoking status, regular exercise, and body mass index.

During the 6,699,462 person-years of follow-up, 109,239 subjects developed MetS (16.3 per 1000 person-years). The cumulative incidence rate of MetS was highest in the NAFLD group (FLI ≥ 60, incidence rate of 158.3 per 1000 person-years), followed by the borderline NAFLD group (30 ≤ FLI < 60, incidence rate of 91.4 per 1000 person-years), and the control group (FLI < 30, incidence rate of 13.3 per 1000 person-years) (Table 2). Moreover, the risk of MetS was positively associated with FLI in a dose-dependent manner when classified into ten groups according to the FLI index (Figure 2).

The risk for each metabolic component of MetS showed similar trends to the risk of MetS, even after adjusting for confounding variables (Supplementary Figure S1 and Supplementary Table S1). All the metabolic components were positively associated with FLI in a dose-dependent manner.

### 3.3. Stratified Analysis According to Various Subgroups

We performed stratified analysis by various factors including age, sex, alcohol consumption, smoking status, regular exercise, obesity, and dyslipidemia. Stratified analyses by baseline variables showed that the associations between high FLI and increased risk of MetS were consistent in all strata regardless of baseline characteristics (Supplementary Table S2). In particular, the strength of the association between FLI and MetS was stronger in subjects who were ≥30 years old, male, alcohol consumers, current smokers, and regular exercisers. The increased risk of incident MetS was attenuated in obese (BMI ≥ 25) subjects or those with dyslipidemia compared to non-obese subjects or those without dyslipidemia.

## 4. Discussion

Through this nationwide population-based cohort study, we found that NAFLD is associated with a higher risk of MetS in adults aged 20 to 39 years, providing clinical evidence for an association between NAFLD and MetS in young adults. In addition, subjects with NAFLD have a higher risk for all the metabolic components consisting MetS.

This study is significant in that it demonstrated NAFLD can precede MetS in young adults through a sufficient follow-up period in a large cohort. Several longitudinal studies have reported that NAFLD could be a risk factor for MetS. However, these studies have limitations such as a small number of patients or NAFLD defined as an elevation of alanine aminotransferase or gamma-glutamyltransferase [10,11,12,25]. This is the first study to reveal that the degree of hepatic steatosis is a leading risk factor for the occurrence of MetS in a population-base of young adults.

The first mechanism to explain that fatty liver is a precursor of MetS regards the pattern of lipid partitioning and insulin resistance [12,26,27]. When fat is deposited in insulin-sensitive organs such as the liver, muscle, and visceral compartments, free fatty acids and inflammatory cytokines increase, while adiponectin levels decrease [28,29]. In this case, peripheral insulin resistance and early atherogenesis may develop, which may eventually lead to disturbances in glucose metabolism and MetS [30,31]. Fatty liver, apart from visceral fat, plays a central role in the development of insulin resistance [32]. To some extent, hepatic triacylglycerol (TAG) synthesis acts as an adaptive process when TAG precursors are abundant and allows storage of lipids in their least toxic form. However, when this compensatory limit is exceeded, an excess of fatty acids leads to cytotoxicity and impaired metabolism. Indeed, in a case-control study, serum free fatty acid was significantly higher in patients with NAFLD than healthy controls [33]. Furthermore, this free fatty acid was positively associated with all metabolic parameters [34]. In our study, the level of hepatic steatosis and the risk of MetS were analyzed in a dose-dependent manner, which is interpreted as an increase in insulin resistance as hepatic steatosis increases. Taken together, NAFLD is associated with the future onset of MetS in young adults.

There is still debate about the clinical significance of simple steatosis in a metabolically healthy population [15,35]. Recently the term “metabolic-associated fatty liver disease” has been proposed for patients with fatty liver [36].

The second hypothesis is that the current diagnostic criteria for MetS are insufficient in detecting early insulin resistance, so NAFLD might be diagnosed before MetS. In fact, even in the presence of insulin resistance, fasting glucose will return to normal due to compensatory hyperinsulinemia and adequate pancreatic b-cell reserve [37]. Fasting glucose is an important item in the diagnosis of MetS, and, therefore, the current definition of MetS has the potential to underestimate patients with early insulin resistance [38].

This study is also significant in that it found NAFLD is a precursor of MetS, especially in ‘young’ adults. Contrary to recent reports that MetS in young adults is increasing, MetS-related tests are not frequently conducted on young adults, and MetS is underestimated due to missing data and lack of interest from clinicians [39]. The increase in MetS in young adults is a big social problem, and finding a preventable factor is important for long-term prognosis. In previous studies, excessive abdominal fat and lack of exercise were reported as risk factors for MetS in young people [40]. In this study, NAFLD was also identified as a preventable risk factor. This study was limited to patients without MetS components in the initial screening, and the effect of NAFLD on MetS could be analyzed without other confounding metabolic factors. Therefore, more active lifestyle intervention measures for young adults diagnosed with NAFLD may help prevent MetS and other metabolic diseases in the future.

Finally, this study showed that relatively lean NAFLD may also be associated with MetS. In this study, the average BMI of patients diagnosed with MetS was 23.7 and the proportion of lean NAFLD was 37.9% (defining BMI less than 23, Eastern standard). In lean NAFLD, such as obese NAFLD, insulin resistance acts as an important mechanism, and some reports show that insulin resistance is more severe in lean NAFLD than in obese NAFLD patients [41]. In particular, a greater association between MetS and NAFLD was reported in younger ages with relatively low BMI, which is consistent with our findings [9].

This study has several limitations. One of the major limitations of this study is that we used FLI as definition of NAFLD. FLI was calculated using waist circumstance and triglyceride. However, these two factors are also considered as components of metabolic syndrome. Thus, NAFLD in our study and metabolic syndrome may confound via higher waist circumstance and triglyceride. Moreover, the risk of hypertriglyceridemia and abdominal obesity may have been overestimated in this analysis. However, FLI is one of the well-validated methods, especially in large scale studies, and we think that it can contribute to the overall trend [22,42]. Further research is needed to determine whether the same results will be obtained in the image-defined NAFLD cohort rather than the FLI-defined NAFLD cohort. Second, the screening rate may be slightly lower in young adults compared to those in their 40s or older, which may cause a selection bias problem. Third, we cannot provide information on whether improvement in NAFLD can lower the risk of MetS.

## 5. Conclusions

In conclusion, the degree of hepatic steatosis can predict future MetS occurrence. Therefore, clinical attention with the perspective of long-term prevention of MetS should be given to young adults diagnosed with fatty liver.

## Figures and Tables

**Table 1:** Baseline characteristics of the study population.

Variable	FLI < 30	30 ≤ FLI < 60	FLI ≥ 60	*p*-Value
	(*n* = 1,585,018)	(*n* = 71,642)	(*n* = 2532)	
Age, years	29.2 ± 4.6	31.4 ± 4.0	31.8 ± 3.8	<0.001
20–29 y	880,059 (55.5)	24,361 (34.0)	769 (30.4)	<0.001
30–39 y	704,959 (44.5)	47,281 (66.0)	1763 (69.6)	
Male	686,261 (43.3)	68,680 (95.9)	2452 (96.8)	<0.001
Body mass index, kg/m^2^	21.1 ± 2.4	25.6 ± 1.9	27.8 ± 7.2	<0.001
<18.5	208,844 (13.2)	5 (0.01)	0 (0)	
18.5–23	1,033,206 (65.2)	4672 (6.5)	29 (1.2)	
23–25	247,216 (15.6)	21,440 (29.9)	222 (8.8)	
25–30	94,898 (6.0)	44,233 (61.7)	1974 (78.0)	
≥30	854 (0.05)	1292 (1.8)	307 (12.1)	
Low-income status	234,208 (14.8)	6607 (9.2)	242 (9.6)	<0.001
Dyslipidemia	34,692 (2.19)	5821 (8.1)	338 (13.4)	<0.001
Smoking				<0.001
Non-smoker	1,103,436 (69.6)	23,765 (33.2)	749 (29.6)	
Ex-smoker	120,225 (7.6)	10,752 (15.0)	374 (14.8)	
Current smoker	361,357 (22.8)	37,125 (51.8)	1409 (55.7)	
Alcohol consumption				<0.001
None	705,868 (44.5)	17,595 (24.6)	520 (20.5)	
Mild-to-moderate *	879,150 (55.5)	54,047 (75.4)	2012 (79.5)	
Regular exercise	188,503 (11.9)	10,212 (14.3)	352 (13.9)	<0.001
Waist circumference, cm	71.9 ± 7.1	84.5 ± 3.4	86.3 ± 3.0	<0.001
Systolic blood pressure, mm Hg	110.9 ± 9.1	116.1 ± 7.3	116.7 ± 6.9	<0.001
Diastolic blood pressure, mm Hg	69.6 ± 7.21	73.2 ± 6.3	74.1 ± 6.1	<0.001
Fasting glucose, mg/dL	85.5 ± 7.8	87.4 ± 7.6	87.9 ± 7.9	<0.001
Total cholesterol, mg/dL	176.3 ± 28.6	196.0 ± 34.6	206.1 ± 31.4	<0.001
HDL cholesterol, mg/dL	62.6 ± 19.7	54.6 ± 32.0	55.4 ± 18.5	<0.001
LDL cholesterol, mg/dL	116.2 ± 348.8	124.2 ± 163.5	128.3 ± 67.1	<0.001
Triglyceride, mg/dL	68.4 (68.4–68.5)	112.8 (112.6–113.0)	124.3 (123.5–125.1)	<0.001
Fatty liver index	7.1 ± 6.5	38.9 ± 7.2	65.8 ± 5.3	<0.001

Data are expressed as mean ± standard deviation, geometric mean (95% confidence interval), or number and percentage. Abbreviations: MetS, metabolic syndrome; HDL, high density lipoprotein; LDL, low density lipoprotein. * mild-to-moderate drinking: less than 30 g/day for men, 20 g/day for women.

**Table 2:** Risk of developing metabolic syndrome according to fatty liver index.

	Number	Duration (Person-Year)	Events	Incidence Rate (Per 1000 p-y)	Model 1 *		Model 2 ^†^		Model 3 ^§^	
					IRR (95% CI)	*p*	IRR (95% CI)	*p*	IRR (95% CI)	*p*
Total										
FLI < 30	1,585,018	6,450,322	86,006	13.33	1 (ref)	<0.001	1 (ref)	<0.001	1 (ref)	<0.001
30–60	71,642	242,083	22,116	91.36	6.9 (6.8–7.0)		4.1 (4.1–4.2)		2.1 (2.1–2.2)	
≥60	2532	7056	1117	158.30	11.9 (11.2–12.6)		7.1 (6.7–7.6)		2.9 (2.8–3.1)	

Abbreviations: p-y, person-year; IRR, incidence rate ratio; CI, confidence interval; FLI, fatty liver index. * Model 1: unadjusted model. ^†^ Model 2: adjusted for age, sex. ^§^ Model 3: adjusted for age, sex, alcohol consumption, smoking, regular exercise, body mass index.

## Data Availability

The datasets used and/or analyzed during the current study are available from the corresponding author on reasonable request.

## Supplementary Materials

The following supporting information can be downloaded at: https://www.mdpi.com/article/10.3390/jcm11040935/s1, Figure S1: Fatty liver index and risk of metabolic components by decile groups. Table S1: Risk of metabolic components according to fatty liver index. Table S2: Relationship between fatty liver index and risk of metabolic syndrome stratified by baseline characteristics.
