# Supplementary material for: Nonalcoholic Fatty Liver Disease Is a Precursor of New-Onset Metabolic Syndrome in Metabolically Healthy Young Adults
Source: J Clin Med. 2022 Feb 11;11(4):935. doi: 10.3390/jcm11040935 (PMC8878201; doi:10.3390/jcm11040935)
Supplement: Supplementary file 1 [file jcm-11-00935-s001.zip › jcm-1592129-supplementary.pdf]

Supplementary Table S1. Risk of metabolic components according to fatty liver index

Outcome	FLI	Number	Duration (person-year)	Events, n	Crude incidence rate (per 1,000 p-y)	Model 1*		Model 2†		Model 3§	
						IRR (95% CI)	P	IRR (95% CI)	P	IRR (95% CI)	P
**Low HDL cholesterol**	<30	1585018	5927150	254558	42.95	1(Ref.)	<.0001	1(Ref.)	<.0001	1(Ref.)	<.0001
	30-<60	71642	254518	16813	66.06	1.53 (1.51-1.56)		1.75 (1.73-1.78)		1.34 (1.31-1.36)	
	≥60	2532	8703	571	65.61	1.52 (1.40-1.65)		1.73 (1.60-1.88)		1.18 (1.09-1.28)	
**High triglyceride**	<30	1585018	5677746	331836	58.45	1(Ref.)	<.0001	1(Ref.)	<.0001	1(Ref.)	<.0001
	30-<60	71642	166404	42647	256.29	4.38 (4.34-4.42)		2.64 (2.61-2.67)		2.31 (2.28-2.34)	
	≥60	2532	4680	1743	372.45	6.37 (6.07-6.67)		3.81 (3.64-4.00)		3.15 (3.00-3.31)	
**Elevated fasting glucose**	<30	1585018	5533261	399765	72.25	1(Ref.)	<.0001	1(Ref.)	<.0001	1(Ref.)	<.0001
	30-<60	71642	214766	30574	142.36	1.97 (1.94-1.99)		1.38 (1.36-1.39)		1.21 (1.20-1.23)	
	≥60	2532	6747	1222	181.11	2.50 (2.36-2.65)		1.74 (1.64-1.84)		1.44 (1.36-1.53)	
	<30	1585018	5383924	403287	74.91	1(Ref.)	<.0001	1(Ref.)	<.0001	1(Ref.)	<.0001
	30-<60	71642	177182	39258	221.57	2.95 (2.92-2.98)		1.63 (1.62-1.65)		1.23 (1.22-1.25)	

**Elevated blood pressure**	≥60	2532	5259	1580	300.41	4.01 (3.81-4.21)		2.20 (2.09-2.31)		1.47 (1.40-1.54)	
**Abdominal obesity**	<30	1585018	6232612	160577	25.76	1(Ref.)	<.0001	1(Ref.)	<.0001	1(Ref.)	<.0001
	30-<60	71642	203974	31717	155.50	6.03 (5.96-6.10)		5.21 (5.14-5.28)		1.71 (1.69-1.74)	
	≥60	2532	5388	1551	287.86	11.17 (10.62-11.74)		9.71 (9.24-10.21)		2.27 (2.16-2.39)	

Abbreviations: p-y, person-year; IRR, incidence rate ratio; HDL, high density lipoprotein; LDL, low density lipoprotein; CI, confidence interval; FLI, fatty liver index.

* Model 1: unadjusted model

† Model 2: adjusted for age, sex

§ Model 3: adjusted for age, sex, alcohol drink, smoking, regular exercise, body mass index

Supplementary Table S2. Relationship between fatty liver index and risk of metabolic syndrome stratified by baseline characteristics

Subgroup	FLI	Number	Duration (person-year)	Events	Crude incidence rate (per 1,000 p-y)	Multivariable Model*		
						IRR (95% CI)	P-value	P for interaction
Age								<0.001
	<30	880059	3890818	48836	12.55	1 (reference)	<0.001	
<30	30-<60	24361	93383	9363	100.26	2.05 (1.99-2.10)		
	≥60	769	2493	405	162.47	2.62 (2.37-2.89)		
	<30	704959	2559504	37170	14.52	1 (reference)	<0.001	
≥30	30-<60	47281	148700	12753	85.76	2.24 (2.19-2.30)		
	≥60	1763	4563	712	156.02	3.27 (3.03-3.53)		
Sex								<0.001
	<30	686261	2903037	67403	23.22	1 (reference)	<0.001	
Male	30-<60	68680	233230	21364	91.60	2.34 (2.30-2.39)		
	≥60	2452	6812	1093	160.46	3.36 (3.16-3.57)		
Female	<30	898757	3547286	18603	5.24	1 (reference)	<0.001	

	30-<60	2962	8853	752	84.95	2.83 (2.62-3.06)		
	≥60	80	244	24	98.20	3.09 (2.07-4.62)		
Alcohol consumption								<0.001
None	<30	705868	2780924	28721	10.33	1 (reference)	<0.001	
	30-<60	17595	56029	5223	93.22	2.01 (1.94-2.09)		
	≥60	520	1374	210	152.83	2.81 (2.44-3.22)		
Mild-to-moderate	<30	879150	3669399	57285	15.61	1 (reference)	<0.001	
	30-<60	54047	186054	16893	90.80	2.18 (2.14-2.23)		
	≥60	2012	5682	907	159.62	3.04 (2.84-3.25)		
Smoking								<0.001
Non-smoker	<30	1103436	4465478	38829	8.70	1 (reference)	<0.001	
	30-<60	23765	81433	6740	82.77	2.02 (1.95-2.08)		
	≥60	749	2172	308	141.79	2.79 (2.49-3.12)		
Ex-smoker	<30	120225	499109	8933	17.90	1 (reference)	<0.001	
	30-<60	10752	36478	2900	79.50	2.37 (2.25-2.49)		
	≥60	374	1065	130	122.12	3.08 (2.58-3.68)		

	<30	361357	1485736	38244	25.74	1 (reference)	<0.001	
Current smoker	30-<60	37125	124171	12476	100.47	2.27 (2.21-2.32)		
	≥60	1409	3819	679	177.78	3.27 (3.02-3.53)		
Regular exercise								0.001
	<30	1396515	5685919	72215	12.70	1 (reference)	<0.001	
No	30-<60	61430	208430	18705	89.74	2.13 (2.08-2.17)		
	≥60	2180	6184	951	153.78	2.85 (2.67-3.05)		
	<30	188503	764404	13791	18.04	1 (reference)	<0.001	
Yes	30-<60	10212	33653	3411	101.36	2.06 (1.97-2.16)		
	≥60	352	872	166	190.35	3.23 (2.77-3.78)		
Body mass index, kg/m^2								<0.001
	<30	1489266	6090333	68329	11.22	1 (reference)	<0.001	
<25	30-<60	26117	92641	6167	66.57	3.22 (3.14-3.31)		
	≥60	251	782	71	90.81	4.41 (3.50-5.57)		
≥25	<30	95752	359990	17677	49.10	1 (reference)	<0.001	
	30-<60	45525	149442	15949	106.72	1.93 (1.89-1.98)		

	≥60	2281	6274	1046	166.71	2.99 (2.81-3.19)		
Dyslipidemia								<0.001
	<30	1550326	6320076	82585	13.07	1 (reference)	<0.001	
No	30-<60	65821	223929	20127	89.88	2.09 (2.05-2.13)		
	≥60	2194	6179	955	154.57	2.85 (2.67-3.05)		
	<30	34692	130247	3421	26.27	1 (reference)	<0.001	
Yes	30-<60	5821	18154	1989	109.56	1.79 (1.67-1.92)		
	≥60	338	878	162	184.57	2.50 (2.12-2.96)		

Abbreviations: p-y, person-year; IRR, incidence rate ratio; CI, confidence interval; FLI, fatty liver index.

*Adjusted for age, sex, alcohol consumption, smoking, regular exercise, and body mass index.

The dot represents the incidence rate ratio, and the vertical line represents corresponding 95% confidence interval. The graph was adjusted for age, sex, alcohol consumption, smoking status, regular exercise, and body mass index.

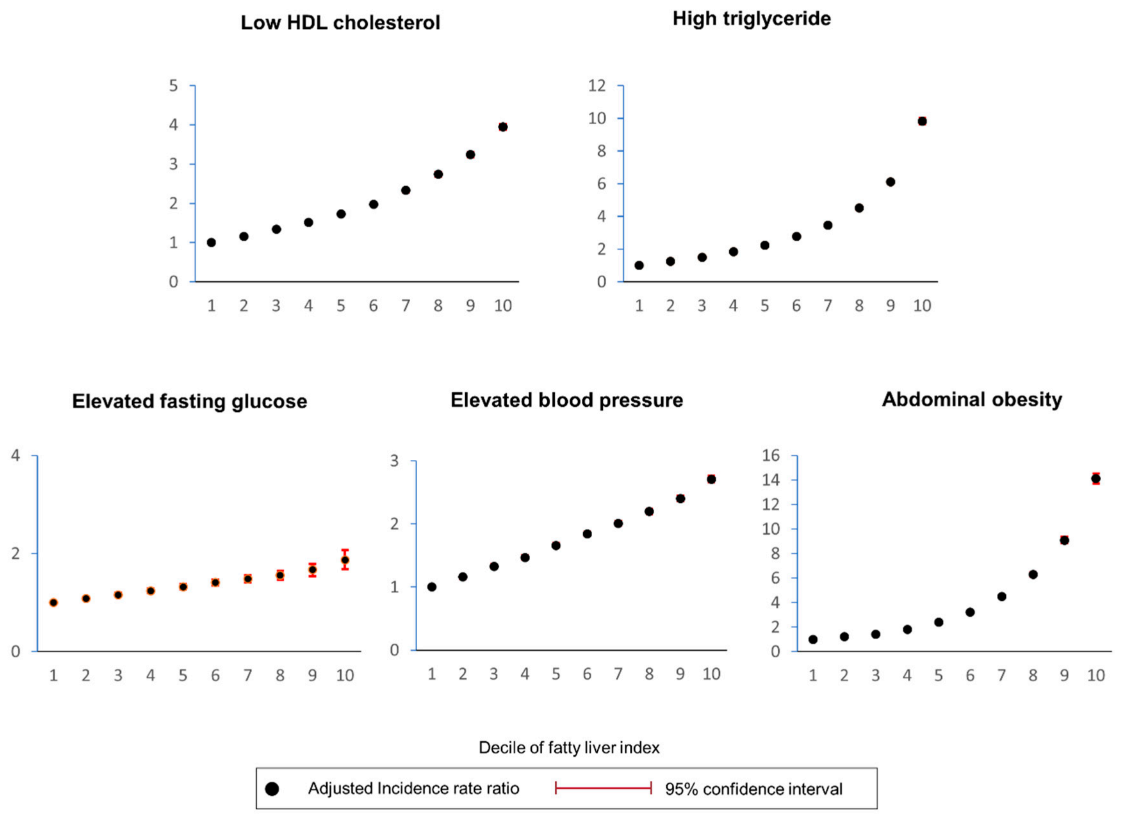

Supplementary Figure S1. Fatty liver index and risk of metabolic components by decile groups
